# Comprehensive Analysis of YTH Domain Family in Lung Adenocarcinoma: Expression Profile, Association with Prognostic Value, and Immune Infiltration

**DOI:** 10.1155/2021/2789481

**Published:** 2021-08-26

**Authors:** Kuan Hu, Lei Yao, Yuanliang Yan, Lei Zhou, Juanni Li

**Affiliations:** ^1^Department of Hepatobiliary Surgery, Xiangya Hospital, Central South University, Changsha, 410008 Hunan, China; ^2^Department of Pharmacy, Xiangya Hospital, Central South University, Changsha, 410008 Hunan, China; ^3^National Clinical Research Center for Geriatric Disorders, Xiangya Hospital, Central South University, Changsha, 410008 Hunan, China; ^4^Department of Anesthesiology, Third Xiangya Hospital of Central South University, Changsha, 410008 Hunan, China; ^5^Department of Pathology, Xiangya Hospital, Central South University, Changsha, 410008 Hunan, China

## Abstract

**Background:**

All YTH domain family members are m^6^A reader proteins accounting for the methylation modulation involved in the process of tumorgenesis and tumor progression. However, the expression profiles and roles of the YTH domain family in lung adenocarcinoma (LUAD) remain to be further illustrated.

**Methods:**

GEPIA2 and TNMplot databases were used to generate the expression profiles of the YTH family. Kaplan-Meier plotter database was employed to analysis the prognostic value of the YTH family. Coexpression profiles and genetic alterations analysis of the YTH family were undertaken using the cBioPortal database. YTH family protein-associated protein-protein interaction (PPI) network was identified by using STRING. Functional enrichment analysis was performed with the help of the WebGestalt database. The correlation analysis between the YTH family and immune cell infiltration in LUAD was administrated by using the TIMER2.0 database.

**Results:**

mRNA expression of YTHDC1 and YTHDC2 was significantly lower in LUAD, whereas YTHDF1, YTHDF2, and YTHDF3 with apparently higher expression. YTHDF2 expression was observed to be the highest in the nonsmoker subgroup, and its expression gradually decreased with the increased severity of smoking habit. LUAD patients with low expression of YTHDC2, YTHDF1, and YTHDF2 were correlated with a better overall survival (OS) time. The YTHDF1 genetic alteration rate was 26%, which was the highest in the YTH family. The major cancer-associated functions of YTH family pointed in the direction of immunomodulation, especially antigen processing and presentation. Most of the YTH family members were significantly correlated with the infiltration of CD4+ T cells, CD8+ T cells, macrophages, and neutrophils, indicating the deep involvement of the YTH domain family in the immune cell infiltration in LUAD.

**Conclusion:**

The molecular and expression profiles of the YTH family were dysregulated in LUAD. YTH family members (especially YTHDC2) were promising biomarkers and potential therapeutic targets that may bring benefit for the patients with LUAD.

## 1. Introduction

As the leading cause of cancer-related deaths in global, lung cancer is composed of two major subtypes lung squamous cell carcinoma (LUSC) and lung adenocarcinoma (LUAD), with LUAD exhibits relatively higher incidence and mortality [1–3]. While tremendous efforts have been made in drug discovery against LUAD, the clinical outcomes of most patients with LUAD remain to be poor [4]. Hence, exploring novel biomarkers and molecular targets is of great value for the development of the LUAD therapeutic strategy.

Dysregulation of RNA methylation has frequently been reported to be implicated in the initiation and progression of cancer [5–7]. N6-Methyladenosine (m6A) modification, as the most common RNA methylation, has received intensive attention these days and is expected to be a promising therapeutic target against cancer [8]. With the function of recognizing m6A-modified mRNA and regulating the expression of target genes, the YT521-B homology (YTH) family as the major reading proteins (known as “readers”) is composed of five proteins that carry the highly conserved YTH domain in common. These proteins are further classified into three categories: YTH domain-containing 1 (YTHDC1), YTH domain-containing 2 (YTHDC2), and YTH m6A-binding protein (YTHDF) including YTHDF1-3. Accumulative studies have shown the robust association between YTH family members and various types of cancer [9]. For instance, there was proved to be a positive correlation between YTHDF1 overexpression and poor prognosis in patients with liver cancer [10]. In addition, the silence of YTHDF2 resulted in the increased invasion of tumor cells in pancreatic cancer [11]. Furthermore, it was reported that YTHDC2 could promote radiotherapy resistance of nasopharyngeal carcinoma via activation of the AKT signal pathway [12]. However, the expression signatures and function of YTH family proteins in LUAD initiation and progression is still lacking.

In the present study, we aimed to further broaden the understanding of the role of the YTH family in LUAD through various public databases, thereby providing novel insights into the diagnosis and treatment of LUAD. The expression profiles, prognostic value, and functional enrichment analysis of YTH family members in LUAD were evaluated, and the correlation of immune cell infiltration with the YTH family was also discussed.

## 2. Materials and Methods

### 2.1. GEPIA2 and TNMplot

Gene Expression Profiling Interactive Analysis (GEPIA) 2.0 and TNMplot are two databases that support comprehensive expression analyses based on TCGA and GTEx data [13–15]. The expression profiles of YTH family members in LAUD and normal lung tissue were retrieved from these two databases. A cutoff of 0.05 in *p* value was set as statistical significance. The relative expression of each YTH in LUAD was compared using GEPIA2. The color density of each block represents the median expression value of each YTH member in LUAD tumor tissue, normalized by the maximum median expression value across all blocks. All the databases used in our study were summarized in Supplementary Table S1.

### 2.2. UALCAN

UALCAN is a portal for tumor subgroup analysis of different gene expressions [16]. In this study, UALCAN database was also applied to analysis the different expressions of YTH family members based on smoking habits. A cutoff of 0.05 in *p* value was set as statistical significance.

### 2.3. Kaplan-Meier Plotter

Kaplan-Meier plotter database is used for evaluating the prognostic role of the expression level of specific gene [17]. In this study, overall survival (OS), first progression (FP), and postprogression survival (PPS) of LUAD patients with different expressions of each YTH family member were compared, respectively, through the Kaplan-Meier plotter database. A cutoff of 0.05 in *p* value was set as statistical significance.

### 2.4. cBioPortal

cBioPortal is a database of cancer genomics which provides download and analysis of genomic alteration data of diverse types of cancer [18, 19]. A dataset of 230 patients with LUAD (TCGA, Firehose Legacy) from cBioPortal was used for the analyses of coexpression and genetic alterations of the YTH family.

### 2.5. STRING and Cytoscape

Probable protein-protein interactions (PPIs) among YTH family members were predicted using STRING, and a database provides an interactive network among interested proteins [20, 21]. In addition, 152 YTH-associated genes were selected from cBioPortal, then, Cytoscape was used to generate the molecular interaction networks [22].

### 2.6. WebGestalt

WebGestalt is a comprehensive tool online that supports gene set enrichment analysis and network topology analysis [23]. Kyoto Encyclopedia of Genes and Genomes (KEGG) pathway and Gene Ontology (GO) enrichment analyses were performed by using WebGestalt to get the enrichment pathways correlated to the YTH family in LUAD.

### 2.7. TIMER2.0

TIMER2.0 is a public resource that offers comprehensive analyses of immune cells infiltration in various types of cancer [24]. Here, we conduct the “immune association” module to obtain the scatterplots which show the association between the expression of YTH family proteins and different types of infiltrated immune cells (CD4+ T cells, CD8+ T cells, B cells, dendritic cells, macrophages, and neutrophils).

## 3. Results

### 3.1. Expression Profiles of YTH Domain Family in Patients with LUAD

**Figure 1:**
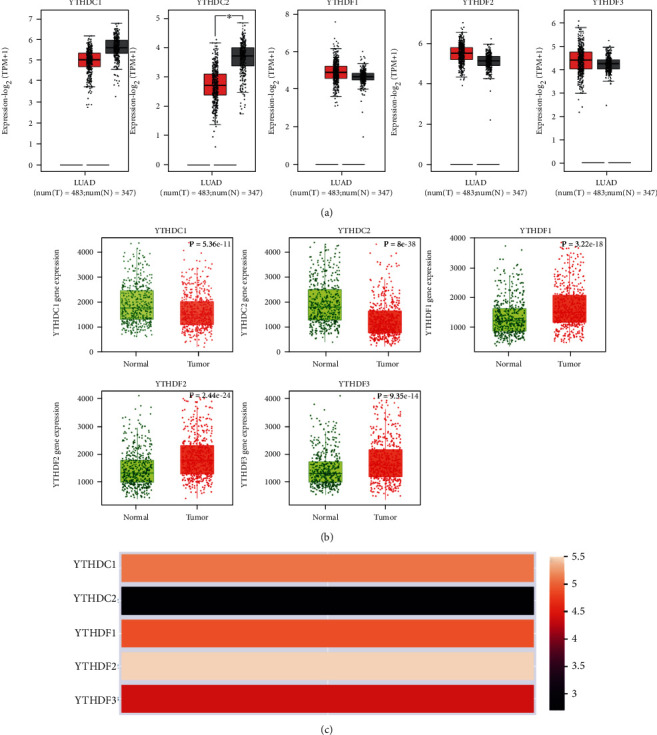
The mRNA expression levels of the YTH family in LUAD were compared with that in normal lung tissues. (a) Data were retrieved from the GEPIA2 database. (b) Data were retrieved from the TNMplot database. (c) The relative expression levels of each YTH family member in LUAD patients (GEPIA2). ^∗^*p* < 0.05.

Data returned from both GEPIA2 and TNMplot databases demonstrated that the mRNA expression of YTH domain family members in LUAD tissues was of widely divergence comparing with that in adjacent normal tissues, especially in the cases of YTHDC2 with significantly lower expression, and YTHDF1, YTHDF2, and YTHDF3 with apparently higher expression only in TNMplot database (Figures 1(a) and 1(b)). Moreover, the expression abundance of each YTH member in LUAD varied. The results showed that the relative expression of YTHDC2 was the lowest in the YTH domain family, while the expression of YTHDF3 was the highest (Figure 1(c)).

**Figure 2:**
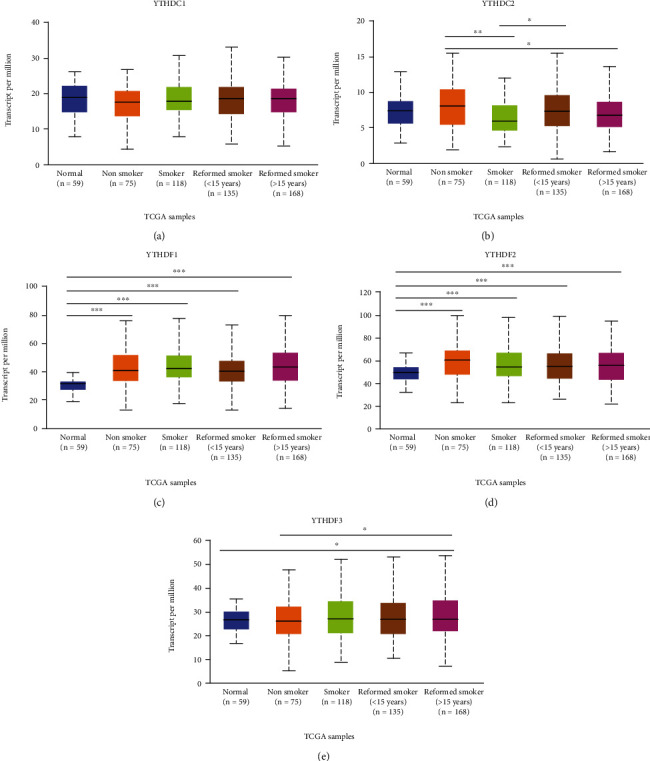
The association between the expression of YTH family members and different smoking habits from UALCAN (nonsmoker, smoker, reformed smoker). ^∗^*p* < 0.05, ^∗∗^*p* < 0.01, ^∗∗∗^*p* < 0.001.

There was no significant difference in the expression of YTH family molecules in the four clinical stage subgroups of LUAD (data no shown). While intriguingly, based on the classification of the status of smoking habits (nonsmoker, smoker, reformed smoker1 (who are current reformed smokers for ≤15 years) and reformed smoker2 (who are current reformed smokers for >15 years)) in LUAD patients, YTHDC2 expression was observed to be the highest in nonsmoker subgroup, and its expression gradually decreased with the increased severity of smoking habit, suggesting that the smoking habits might reflect the expression of YTHDC2 level, and even for the LUAD patient with smoking, smoking cessation can effectively reduce YTHDC2 expression (Figure 2). Moreover, we made the logistic regression analysis using LUAD data from TCGA, and the results showed that the relationship between smoking and YTHDC2 expression was not statistically significant, indicating that more samples are needed to verify the relationship between them (Supplementary Table S3). Furthermore, we analyzed the prognostic effect of YTHDC2 on both smoking and nonsmoking LUAD patients and found that both smoking and nonsmoking patients with high expression of YTHDC2 showed better prognosis (HR = 0.65, *p* = 0.0011; HR = 0.19, *p* = 5.8*e* − 08;, respectively) (Supplementary Figure S1).

### 3.2. YTH Domain Family in Prognosis of LUAD Patients

**Figure 3:**
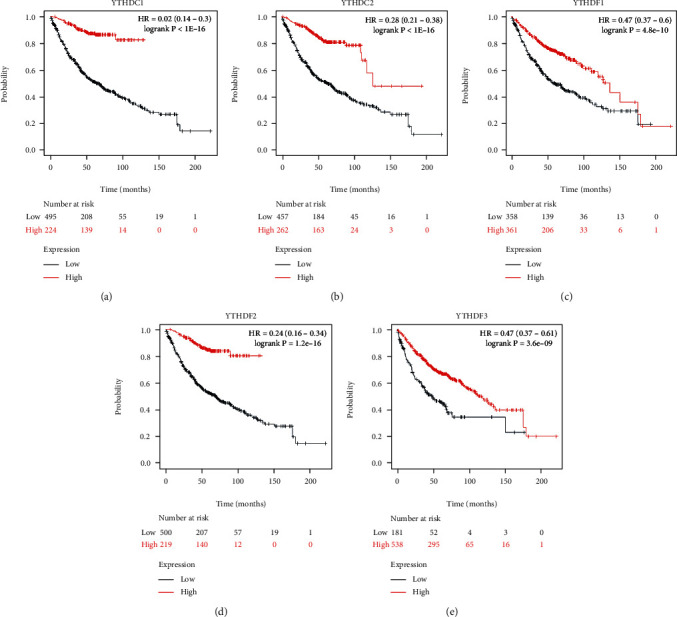
The correlations between the expression of YTH family members and overall survival (OS) of LUAD patients were analyzed through the Kaplan-Meier plotter website.

**Figure 4:**
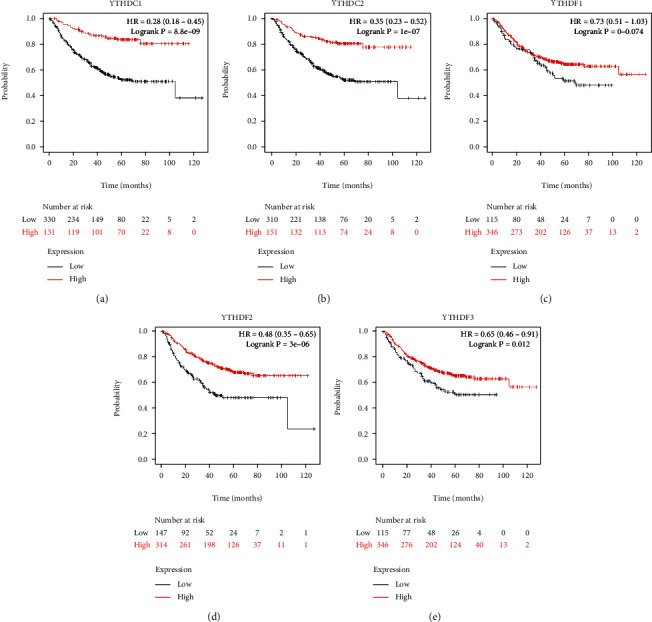
The correlations between the expression of YTH family members and first progression (FP) of LUAD patients were retrieved from the Kaplan-Meier plotter website.

**Figure 5:**
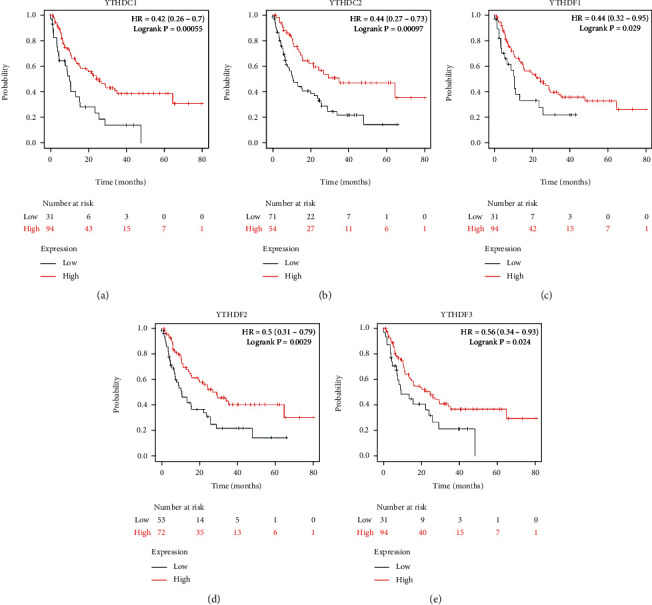
The correlations between the expression of YTH family members and postprogression survival (PPS) of LUAD patients were obtained from the Kaplan-Meier plotter website.

To assess the prognostic value of the YTH domain family in LUAD, the correlations between the expression of YTH family members and survival endpoints like overall survival (OS), first progression (FP), and postprogression survival (PPS) were further analyzed through Kaplan-Meier plotter website. As a consequence, LUAD patients with low expression of YTHDC2 (*p* < 0.001), YTHDF1 (*p* < 0.05), and YTHDF2 (*p* < 0.01) were correlated with a better OS time (Figure 3). Similarly, low expression of YTHDC1 (*p* < 0.001), YTHDC2 (*p* < 0.001), YTHDF2 (*p* < 0.001), and YTHDF3 (*p* < 0.05) were associated with a better FP time (Figure 4). As for the PPS, it was YTHDC1 (*p* < 0.001), YTHDC2 (*p* < 0.001), and YTHDF2 (*p* < 0.005) that linked to a better prognosis (Figure 5).

### 3.3. Genetic Alterations of YTH Domain Family in LUAD

**Figure 6:**
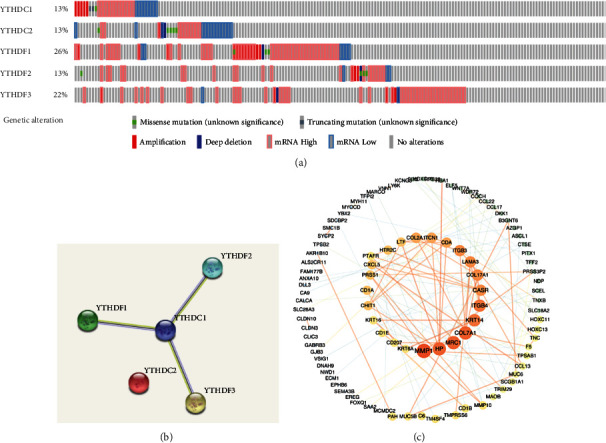
Genetic alterations analysis and molecular interaction analysis of the YTH family in LUAD. (a) Genetic alteration profiles of the YTH family in LUAD by using cBioPortal. (b) The interaction analysis within the YTH family from STRING. (c) The identification of 152 YTH-associated genes which was most frequently altered in LUAD through cBioPortal and Cytoscape.

The frequency and types of genetic alterations in the YTH domain family in LUAD were obtained through the TCGA database and cBioPortal tool. As presented in Figure 6(a), the YTHDF1 genetic alteration rate was 26%, which was the highest in the YTH family. Genetic alteration rates of the other family members are 13% (for YTHDC1, YTHDC2, and YTHDF2) and 22% (YTHDF3). In the aspect of types of genetic alterations, gene amplification, missence mutation, truncating mutation, and mRNA high/low were the main genetic alterations in YTHDC1, YTHDF1, and YTHDF2. Whereas barely missence mutation was observed in YTHDC2, and scarcely missence and truncating mutation were found in YTHDF3 (Figure 6(a)).

### 3.4. Interactive Network Analyses of YTH Domain Family and Associated Molecules

The interactive network of the YTH domain family generated by the STRING database demonstrated that YTHDF1, YTHDF2, and YTHDF3 were concordantly interacted with YTHDC1, revealing YTHDC1 acted as the hub node in this interactive network (Figure 6(b)). However, YTHDC2 was relatively dissociated from its family members in terms of the functional network of interactive molecules. Furthermore, 152 most frequent genes with changed expression level and concomitantly with close correlation to YTH domain family were identified from the cBioPortal database (Supplementary Table S2). Then, the interactive network of these 152 genes was established, which further suggested that molecules including MMP1, HP, MRC1, COL7A1, KRT14, ITGB4, CASR, and COL17A1 may serve as hub genes participating in the biological processes of the YTH domain family in LUAD (Figure 6(c)).

**Figure 7:**
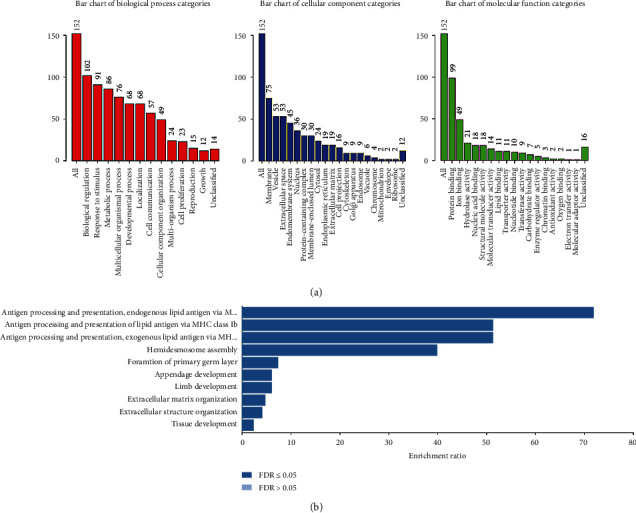
The biological pathway analysis of YTH family from WebGestalt database. (a) The biological process, cellular component, and molecular function which were associated with the YTH family were shown on the bar charts through Gene Ontology (GO) enrichment analysis. (b) Kyoto Encyclopedia of Genes and Genome (KEGG) enrichment analysis of YTH family.

To further understand what kinds of functions are induced by the YTH domain family, the functional enrichment analysis was performed on the basis of the 152 YTH-associated genes by using the WebGestalt database. Consequently, YTH domain family members were mainly enriched in biological functions such as biological regulation, response to stimulus, metabolic process, multicellular organismal process, and developmental process. Moreover, YTH domain family members were also found to be highly enriched in the following cellular component, including membrane, vesicle, extracellular space, endomembrane system, nucleus, protein-containing complex, and membrane-enclosed lumen, and so on. As for the enrichment of molecular function, protein binding, ion binding, hydrolase activity, nucleic acid binding, structural molecule activity, and molecular transducer activity are the top ones with the highest enrichment (Figure 7(a)). In addition, Kyoto Encyclopedia of Genes and Genomes (KEGG) pathway was used to exhibit the enrichment ratio of specific biological functions that contribute to the LUAD development. The top-ranked YTH-associated biological functions involved in the LUAD development were antigen processing and presentation, hemidesmosome assembly, formation of primary germ layer, and appendage development (Figure 7(b)).

### 3.5. Correlation between Immune Cell Infiltration and Each YTH Domain Family Member

**Figure 8:**
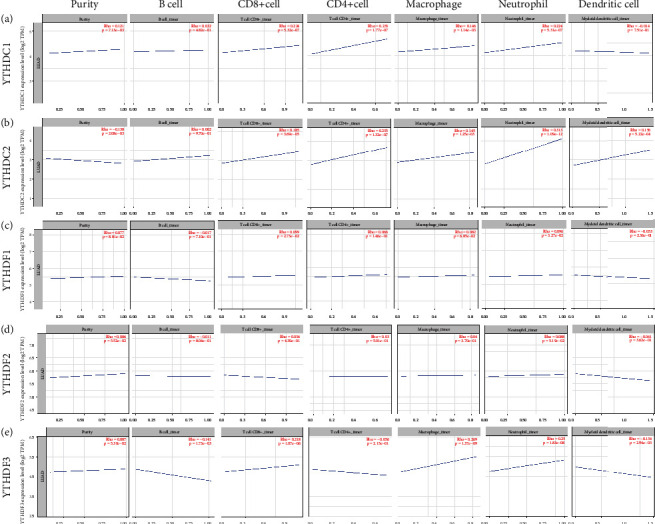
The correlation between immune cell infiltration and each YTH domain family member. The TIMER2.0 database was applied to explore the role of (a) YTHDC1, (b) YTHDC2, (c) YTHDF1, (d) YTHDF2, and (e) YTHDF3 in different immune cell infiltration (CD4+ T cells, CD8+ T cells, B cells, dendritic cells, macrophages, and neutrophils) around LUAD.

Immune cell infiltration in tumor is an indispensable component of tumor microenvironment and an independent index that reflects prognosis and lymphatic metastasis status [25–27]. Thus, we use the TIMER2.0 database to further illustrate the correlation between immune cell infiltration and each YTH domain family members. YTHDC1 expression was significantly correlated with infiltration of several types of immune cells ranging from macrophage (Rho = 0.146, *p* = 1.14 × 10^–3^) to neutrophil (Rho = 0.224, *p* = 5.31 × 10^–7^), CD4+ T cell (Rho = 0.233, *p* = 1.77 × 10^–7^), and CD8+ T cell (Rho = 0.224, *p* = 5.32 × 10^–7^) (Figure 8(a)). YTHDC2 expression was significantly associated with infiltration of macrophage (Rho = 0.145, *p* = 1.25 × 10^–3^), neutrophil (Rho = 0.313, *p* = 1.09 × 10^–12^), CD4+ T cell (Rho = 0.235, *p* = 1.32 × 10^–7^), CD8+ T cell (Rho = 0.185, *p* = 3.69 × 10^–5^), and dendritic cell (Rho = 0.146, *p* = 5.22 × 10^–4^) (Figure 8(b)). In addition, in the cases of YTHDF1, YTHDF2, and YTHDF3, various immune infiltration signatures were observed to have a significant correlation (Figures 8(c)–8(e)). These data indicated the deep involvement of the YTH domain family in the immune cell infiltration in LUAD.

## 4. Discussion

RNA methylation is a vital posttranscriptional modification that participates in various human biological processes [28]. Recent researches on m6A RNA methylation have revealed its facilitating role in the initiation and development of various types of cancer, thus, has gradually become a new direction in oncology research and targeted drug development [29, 30]. All of the five YTH domain family members are m6A reader proteins—the key enzyme that regulates the methylation of target RNA by specific combination with m6A-containing mRNA. With huge potential value, there are multiple studies reporting the role of YTH family proteins in various cancer types. For instance, YTHDF1 was proved to be an oncogene in hepatocellular carcinoma (HCC) owing to its overexpression in HCC patients and association with poor prognosis [10, 31]. The oncogenic role of YTHDF1 may be achieved by the mechanisms of Snail-induced EMT [32] and m6A-dependent activation of the WNT/*β*-catenin pathway [33]. However, the role of YTHDF2 in HCC was found to be contradictory, which acted as either oncogene in a m6A-dependent manner or tumor suppressor gene through EGFR and ERK/MAPK pathway [34, 35]. In the case of ovarian cancer, YTHDF1 was also found to be overexpressed and associated with poor clinical outcome. YTHDF1 accelerated the growth and metastasis of ovarian cancer in vivo and in vitro by promoting the m6A-modified translation of EIF3C [36]. In the field of lung cancer, the expression of YTHDF2 was aberrantly higher and facilitated the proliferation and growth of cancer cells, ribose-5-phosphate, and NADPH induced by pentose phosphate pathway might be the mechanism underlies [37]. YTHDF1 was demonstrated to promote proliferation of cancer cells in nonsmall cell lung cancer (NSCLC), whereas paradoxically, better prognosis and chemotherapy sensitivity were correlated with high YTHDF1 expression and implied the complicated and multiple mechanisms lurking beneath the phenomena observed above [38].

According to our knowledge, a study that systematically focused on the expression, prognostic value, and pathophysiological function of the YTH domain family in LUAD is still lacking. Hence, we preliminarily explore the expression profiles of the YTH domain family and found that only the expression of YTHDC2 is significantly lower in all of the LUAD datasets included in this study, while YTHDF1, YTHDF2, and YTHDF3 are proved to be significantly upregulated only in one dataset. Undoubtedly, YTHDC2 expression is more convincing because data retrieved from multiple datasets showed high concordance. These indicated that YTHDC2 might be the potential tumor suppressor gene. YTHDF1, YTHDF2, and YTHDF3 might as well act as the potential oncogenes with a lower level of evidence.

Then, we explored whether the prognostic value of YTH proteins was in line with their expression trend. Only YTHDC1 passed this round of screening cause its lower expression was correlated with poorer prognosis with statistical significance when using OS, FP, and PPS as the endpoints of survival. While higher expression of YTHDF1, YTHDF2, and YTHDF3 was associated with better clinical outcome, which is in contradiction with their expression profiles in LUAD compared with normal lung tissue. These results pushed YTHDC2 to the foreground as a novel potential target gene against LUAD. Of course, more solid evidence from experiments is needed.

To date, the role of YTHDF2 in lung cancer (especially in LUAD) remains to be further explored. YTHDC2 can promote 6PGD mRNA translation in lung cancer cells by means of m6A modification. We made a horizontal comparison of YTHDC2 in different types of cancer. Unlike the low expression of YTHDC2 in LUAD, YTHDC2 expression was increased in liver cancer and positively related to tumor malignancy [39]. Whereas opposite evidence of YTHDC2 as a tumor suppressor gene is also reported in liver cancer [35], covering the underneath mechanisms a heterogeneous veil. In addition, YTHDC2 exerts a promoting role in the colorectal cancer metastasis through the hypoxia/HIF-1*α*/Twist1 signaling pathway [40]. Our study also for the first time suggested that smoking habits may negatively reflect the expression of YTHDC2 level in LAUD patients. Further investigations based on it may have the opportunities to make breakthrough in targeted drug development and precision medicine of LAUD with smoking habit.

The functional enrichment analysis [41] in this study screens out the biological process and functional interpretation of genes around the YTH family, which further broaden our understanding of the role of the YTH family in the pathophysiological process of LUAD. The major functions of the YTH family seem to point in the direction of immunomodulation, especially antigen processing and presentation. This was in accordance with the finding of a published paper, showing that gene signature of antigen processing and presentation machinery may predict the therapeutic effect of immune checkpoint inhibitors such as anti-PD-1/PD-L1 [42]. Another study gave a similar conclusion that impaired antigen processing and presentation may account for the acquired resistance of immune checkpoint inhibitors and thus led to treatment failure [43] in lung cancer. Therefore, the relationship between the YTH family and antigen processing and presentation in LUAD is worthy of further exploration.

m6A methylation has been reported to play essential roles in tumor immunity. Furthermore, profiles of immune cells infiltration in tumor could act as novel biomarkers that effectively improved the diagnosis and prognosis of many types of cancer [44, 45]. Our study found that in LUAD, most of the YTH family members were significantly correlated with the infiltration of CD4+ T cells, CD8+ T cells, macrophages, and neutrophils. A previous study presented that CD4+ T cells and CD8+ T cells were generally believed to control cancer outcome, while macrophages and neutrophils produce various factors that induce inflammation and stimulus tumor progression [46]. Taken together, the abnormal expression of YTH family members (especially YTHDC2) may alter the profiles of immune cell infiltration in LUAD through some kinds of specific mechanisms, thus influence the clinical outcome and the therapeutic effect of immunotherapy.

However, there were several limitations in our work. Our analysis in this study was mostly based on the data from the online databases, and further laboratory experiments were needed to verify these conclusions. Moreover, further research into the specific molecular mechanisms and molecules interactions would be needed in the future.

## 5. Conclusions

In conclusion, the molecular and expression profiles of the YTH family were dysregulated in LUAD. YTH family members (especially YTHDC2) are promising biomarkers and potential therapeutic targets that may bring benefit for patients with LUAD.

## Data Availability

The original contributions presented in the study are included in the article/Supplementary Material, and further inquiries can be directed to the corresponding authors.

## Supplementary Materials

Supplementary 1 Supplementary Figure S1: the prognostic effect of YTHDC2 on smoking and nonsmoking patients with LUAD using Kaplan-Meier plotter.

Supplementary 2 Supplementary Table S1: summary of bioinformatics databases used in this study for analyzing the role of the YTH domain family in LUAD.

Supplementary 3 Supplementary Table S2: the YTH domain family associated coexpressed genes in LUAD.

Supplementary 4 Supplementary Table S3: the association between the expression of individual YTH family members and clinical characters in LUAD patients.

## References

[1] Imyanitov E. N., Iyevleva A. G., Levchenko E. V. (2021). Molecular testing and targeted therapy for non-small cell lung cancer: current status and perspectives. *Critical Reviews in Oncology/Hematology*.

[2] Zhu K., Chen L., He C. (2020). Prediction of pleural invasion in challenging non-small-cell lung cancer patients using serum and imaging markers. *Disease Markers*.

[3] Chen Z. Q., Huang L. S., Zhu B. (2018). Assessment of seven clinical tumor markers in diagnosis of non-small-cell lung cancer. *Disease Markers*.

[4] Schenk E. L., Patil T., Pacheco J., Bunn P. A. (2021). 2020 innovation-based optimism for lung cancer outcomes. *The Oncologist*.

[5] Sun T., Wu R., Ming L. (2019). The role of m6A RNA methylation in cancer. *Biomedicine & Pharmacotherapy*.

[6] Yan Y., Xu Z., Li Z., Sun L., Gong Z. (2017). An insight into the increasing role of lncRNAs in the pathogenesis of gliomas. *Frontiers in Molecular Neuroscience*.

[7] Xu Z., Yan Y., Qian L., Gong Z. (2017). Long non-coding RNAs act as regulators of cell autophagy in diseases (review). *Oncology Reports*.

[8] Jaffrey S. R., Kharas M. G. (2017). Emerging links between m6A and misregulated mRNA methylation in cancer. *Genome Medicine*.

[9] Panneerdoss S., Eedunuri V. K., Yadav P. (2018). Cross-talk among writers, readers, and erasers of m6A regulates cancer growth and progression. *Science Advances*.

[10] Zhao X., Chen Y., Mao Q. (2018). Overexpression of YTHDF1 is associated with poor prognosis in patients with hepatocellular carcinoma. *Cancer Biomarkers*.

[11] Chen J., Sun Y., Xu X. (2017). YTH domain family 2 orchestrates epithelial-mesenchymal transition/proliferation dichotomy in pancreatic cancer cells. *Cell Cycle*.

[12] He J. J., Li Z., Rong Z. X. (2020). m6A reader YTHDC2 promotes radiotherapy resistance of nasopharyngeal carcinoma via activating IGF1R/AKT/S6 signaling axis. *Frontiers in Oncology*.

[13] Tang Z., Kang B., Li C., Chen T., Zhang Z. (2019). GEPIA2: an enhanced web server for large-scale expression profiling and interactive analysis. *Nucleic Acids Research*.

[14] Bartha Á., Győrffy B. (2021). TNMplot.com: a web tool for the comparison of gene expression in normal, tumor and metastatic tissues. *International Journal of Molecular Sciences*.

[15] Li J., Hu K., He D., Zhou L., Wang Z., Tao Y. (2020). Prognostic value of PLXND1 and TGF-*β*1 coexpression and its correlation with immune infiltrates in hepatocellular carcinoma. *Frontiers in Oncology*.

[16] Chandrashekar D. S., Bashel B., Balasubramanya S. A. H. (2017). UALCAN: a portal for facilitating tumor subgroup gene expression and survival analyses. *Neoplasia*.

[17] Gyorffy B., Lánczky A., Szállási Z. (2012). Implementing an online tool for genome-wide validation of survival-associated biomarkers in ovarian-cancer using microarray data from 1287 patients. *Endocrine-Related Cancer*.

[18] Gao J., Aksoy B. A., Dogrusoz U. (2013). Integrative analysis of complex cancer genomics and clinical profiles using the cBioPortal. *Science Signaling*.

[19] Li J., Hu K., Zhou L. (2020). Spectrum of mesenchymal-epithelial transition aberrations and potential clinical implications: insights from integrative pancancer analysis. *Frontiers in Oncology*.

[20] Szklarczyk D., Morris J. H., Cook H. (2017). The STRING database in 2017: quality-controlled protein-protein association networks, made broadly accessible. *Nucleic Acids Research*.

[21] Szklarczyk D., Gable A. L., Nastou K. C. (2021). The STRING database in 2021: customizable protein-protein networks, and functional characterization of user-uploaded gene/measurement sets. *Nucleic Acids Research*.

[22] Doncheva N. T., Morris J. H., Gorodkin J., Jensen L. J. (2019). Cytoscape StringApp: network analysis and visualization of proteomics data. *Journal of Proteome Research*.

[23] Liao Y., Wang J., Jaehnig E. J., Shi Z., Zhang B. (2019). WebGestalt 2019: gene set analysis toolkit with revamped UIs and APIs. *Nucleic Acids Research*.

[24] Li T., Fu J., Zeng Z. (2020). TIMER2.0 for analysis of tumor-infiltrating immune cells. *Nucleic Acids Research*.

[25] Sun W., Shi H., Yuan Z. (2020). Prognostic value of genes and immune infiltration in prostate tumor microenvironment. *Frontiers in Oncology*.

[26] Wang H. C., Chan L. P., Cho S. F. (2019). Targeting the immune microenvironment in the treatment of head and neck squamous cell carcinoma. *Frontiers in Oncology*.

[27] Liao H., Chen W., Dai Y. (2019). Expression of programmed cell death-ligands in hepatocellular carcinoma: correlation with immune microenvironment and survival outcomes. *Frontiers in Oncology*.

[28] Roundtree I. A., Evans M. E., Pan T., He C. (2017). Dynamic RNA modifications in gene expression regulation. *Cell*.

[29] Lan Q., Liu P. Y., Bell J. L. (2021). The emerging roles of RNA m6A methylation and demethylation as critical regulators of tumorigenesis, drug sensitivity, and resistance. *Cancer Research*.

[30] Li W., Liu J., Ma Z., Zhai X., Cheng B., Zhao H. (2021). m6A RNA methylation regulators elicit malignant progression and predict clinical outcome in hepatocellular carcinoma. *Disease Markers*.

[31] Zhou Y., Yin Z., Hou B. (2019). Expression profiles and prognostic significance of RNA N6-methyladenosine-related genes in patients with hepatocellular carcinoma: evidence from independent datasets. *Cancer Management and Research*.

[32] Lin X., Chai G., Wu Y. (2019). RNA m6A methylation regulates the epithelial mesenchymal transition of cancer cells and translation of Snail. *Nature Communications*.

[33] Liu X., Qin J., Gao T. (2020). YTHDF1 facilitates the progression of hepatocellular carcinoma by promoting FZD5 mRNA translation in an m6A-dependent manner. *Molecular Therapy-Nucleic Acids*.

[34] Zhang C., Huang S., Zhuang H. (2020). YTHDF2 promotes the liver cancer stem cell phenotype and cancer metastasis by regulating OCT4 expression via m6A RNA methylation. *Oncogene*.

[35] Zhong L., Liao D., Zhang M. (2019). YTHDF2 suppresses cell proliferation and growth via destabilizing the EGFR mRNA in hepatocellular carcinoma. *Cancer Letters*.

[36] Liu T., Wei Q., Jin J. (2020). The m6A reader YTHDF1 promotes ovarian cancer progression via augmenting EIF3C translation. *Nucleic Acids Research*.

[37] Sheng H., Li Z., Su S. (2020). YTH domain family 2 promotes lung cancer cell growth by facilitating 6-phosphogluconate dehydrogenase mRNA translation. *Carcinogenesis*.

[38] Shi Y., Fan S., Wu M. (2019). YTHDF1 links hypoxia adaptation and non-small cell lung cancer progression. *Nature Communications*.

[39] Yang Z., Li J., Feng G. (2017). MicroRNA-145 modulates *N*^6^-methyladenosine levels by targeting the 3′-untranslated mRNA region of the *N*^6^-methyladenosine binding YTH domain family 2 protein. *The Journal of Biological Chemistry*.

[40] Tanabe A., Tanikawa K., Tsunetomi M. (2016). RNA helicase YTHDC2 promotes cancer metastasis via the enhancement of the efficiency by which *HIF-1 α* mRNA is translated. *Cancer Letters*.

[41] Gene Ontology Consortium (2019). The gene ontology resource: 20 years and still GOing strong. *Nucleic Acids Research*.

[42] Thompson J. C., Davis C., Deshpande C. (2020). Gene signature of antigen processing and presentation machinery predicts response to checkpoint blockade in non-small cell lung cancer (NSCLC) and melanoma. *Journal for Immunotherapy of Cancer*.

[43] Gettinger S., Choi J., Hastings K. (2017). Impaired HLA class I antigen processing and presentation as a mechanism of acquired resistance to immune checkpoint inhibitors in lung cancer. *Cancer Discovery*.

[44] Zhou R., Zhang J., Zeng D. (2019). Immune cell infiltration as a biomarker for the diagnosis and prognosis of stage I-III colon cancer. *Cancer Immunology, Immunotherapy*.

[45] Klemm F., Maas R. R., Bowman R. L. (2020). Interrogation of the microenvironmental landscape in brain tumors reveals disease-specific alterations of immune cells. *Cell*.

[46] Wang Y., Dong J., Quan Q. (2020). Immune cell infiltration of the primary tumor microenvironment predicted the treatment outcome of chemotherapy with or without bevacizumab in metastatic colorectal cancer patients. *Frontiers in Oncology*.

