# Supplementary material for: Comprehensive Analysis of YTH Domain Family in Lung Adenocarcinoma: Expression Profile, Association with Prognostic Value, and Immune Infiltration
Source: Dis Markers. 2021 Aug 26;2021:2789481. doi: 10.1155/2021/2789481 (PMC8420974; doi:10.1155/2021/2789481)
Supplement: Supplementary 1 — Supplementary Figure S1: the prognostic effect of YTHDC2 on smoking and nonsmoking patients with LUAD using Kaplan-Meier plotter. [file 2789481.f1.docx]

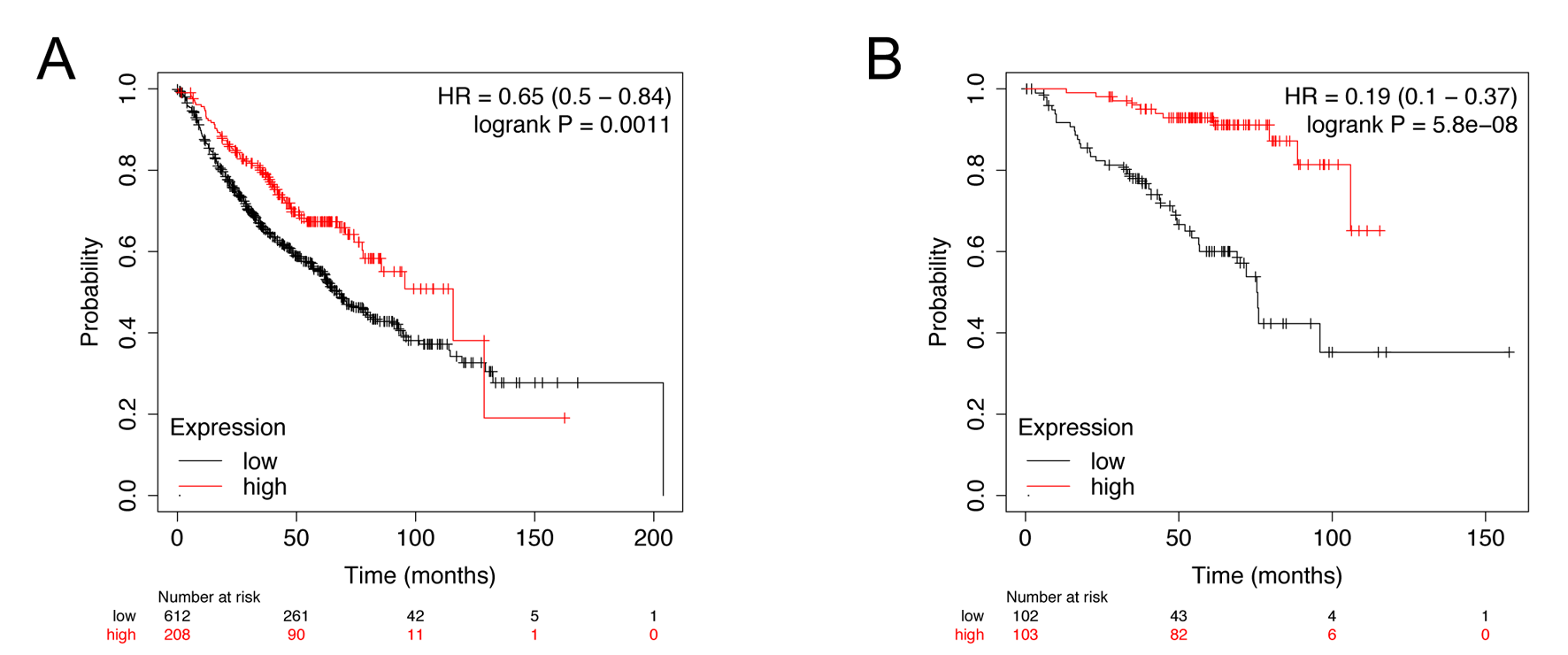


**Supplementary Figure S1.** The prognostic effect of YTHDC2 on smoking and non-smoking patients with LUAD using Kaplan-Meier plotter.
