# Supplementary material for: Comprehensive Analysis of YTH Domain Family in Lung Adenocarcinoma: Expression Profile, Association with Prognostic Value, and Immune Infiltration
Source: Dis Markers. 2021 Aug 26;2021:2789481. doi: 10.1155/2021/2789481 (PMC8420974; doi:10.1155/2021/2789481)
Supplement: Supplementary 2 — Supplementary Table S1: summary of bioinformatics databases used in this study for analyzing the role of the YTH domain family in LUAD. [file 2789481.f2.docx]

| **Databases** | **Authors** | **Samples** | **Homepage links** |
| --- | --- | --- | --- |
| GEPIA2 | Tang Z. et al. | Tissues | http://gepia.cancer-pku.cn/ |
| TNMplot | Bartha Á. et al. | Tissues | https://tnmplot.com/ |
| UALCAN | Chandrashekar DS. et al. | Tissues | http://ualcan.path.uab.edu/index.html |
| Kaplan-Meier plotter | Gyorffy B. et al. | Tissues | http://kmplot.com/analysis/ |
| cBioPortal | Cerami E. et al. | Tissues | http://www.cbioportal.org/ |
| Cytoscape | Doncheva NT et al. | - | - |
| STRING | Szklarczyk D et al. | - | https://string-db.org/ |
| WebGestalt | Liao Y. et al. | - | http://webgestalt.org/ |
| TIMER2.0 | Li T. et al. | Tissues | https://cistrome.shinyapps.io/timer/ |

**Supplementary Table S1.** Summary of bioinformatics databases used in this study for analyzing the role of the YTH domain family in LUAD.
