# Supplementary material for: Comprehensive Analysis of YTH Domain Family in Lung Adenocarcinoma: Expression Profile, Association with Prognostic Value, and Immune Infiltration
Source: Dis Markers. 2021 Aug 26;2021:2789481. doi: 10.1155/2021/2789481 (PMC8420974; doi:10.1155/2021/2789481)
Supplement: Supplementary 3 — Supplementary Table S2: the YTH domain family associated coexpressed genes in LUAD. [file 2789481.f3.docx]

**Supplementary Table S2:** The YTH domain family associated co-expressed genes in LUAD.

| **Gene** | **Log Ratio** | **p-Value** | **expression** |
| --- | --- | --- | --- |
| MCMDC2 | 0.84 | 9.72E-11 | Altered group |
| ACTG1P25 | 1.09 | 2.69E-08 | Altered group |
| EPHB6 | -1.07 | 1.95E-07 | Unaltered group |
| MAOB | -0.84 | 1.06E-06 | Unaltered group |
| PTAFR | -0.89 | 1.15E-06 | Unaltered group |
| ABLIM3 | -0.9 | 1.23E-06 | Unaltered group |
| CCL22 | -0.9 | 5.09E-06 | Unaltered group |
| WNT7A | -1.44 | 5.16E-06 | Unaltered group |
| COL17A1 | -1.69 | 8.05E-06 | Unaltered group |
| ITGB4 | -0.88 | 9.18E-06 | Unaltered group |
| ANXA8 | -1.4 | 9.36E-06 | Unaltered group |
| SIGLEC6 | -0.91 | 9.84E-06 | Unaltered group |
| PCDHGA10 | -1 | 1.05E-05 | Unaltered group |
| UNC5A | -0.86 | 1.23E-05 | Unaltered group |
| LARGE2 | 0.91 | 1.59E-05 | Altered group |
| SEMA3B | -0.95 | 1.59E-05 | Unaltered group |
| VILL | -1.04 | 2.14E-05 | Unaltered group |
| MYH11 | -0.91 | 2.61E-05 | Unaltered group |
| SH3RF2 | -1.08 | 2.66E-05 | Unaltered group |
| TNS4 | -1.45 | 3.01E-05 | Unaltered group |
| SLC28A3 | -1.13 | 3.05E-05 | Unaltered group |
| SPTLC3 | -0.84 | 3.06E-05 | Unaltered group |
| TPSAB1 | -0.85 | 3.13E-05 | Unaltered group |
| SULT1B1 | -0.89 | 3.50E-05 | Unaltered group |
| HR | -0.93 | 3.70E-05 | Unaltered group |
| ECM1 | -0.87 | 4.13E-05 | Unaltered group |
| FOXQ1 | -1.16 | 4.14E-05 | Unaltered group |
| KCNG3 | 0.93 | 5.77E-05 | Altered group |
| ISM1 | -0.81 | 7.02E-05 | Unaltered group |
| AHNAK2 | -0.95 | 7.62E-05 | Unaltered group |
| ITGB3 | -0.91 | 7.70E-05 | Unaltered group |
| MRC1 | -0.84 | 8.21E-05 | Unaltered group |
| CHI3L2 | -0.92 | 8.38E-05 | Unaltered group |
| CCL13 | -0.98 | 9.50E-05 | Unaltered group |
| SYCP2 | 0.97 | 1.04E-04 | Altered group |
| TPSB2 | -0.84 | 1.14E-04 | Unaltered group |
| MYOCD | -0.86 | 1.18E-04 | Unaltered group |
| TRIM29 | -1.5 | 1.24E-04 | Unaltered group |
| SLC6A14 | -1.11 | 1.34E-04 | Unaltered group |
| LY6K | 1.43 | 1.36E-04 | Altered group |
| TNXB | -0.82 | 1.39E-04 | Unaltered group |
| LILRA4 | -0.82 | 1.40E-04 | Unaltered group |
| CDA | -1.17 | 1.44E-04 | Unaltered group |
| FIBCD1 | -0.99 | 1.45E-04 | Unaltered group |
| NDP | -1.08 | 1.49E-04 | Unaltered group |
| CCL17 | -1.03 | 1.66E-04 | Unaltered group |
| FAM177B | -1.21 | 1.74E-04 | Unaltered group |
| STAC | -0.86 | 1.87E-04 | Unaltered group |
| SDK2 | -0.81 | 2.00E-04 | Unaltered group |
| CLDN3 | 0.84 | 2.11E-04 | Altered group |
| TCN1 | -1.79 | 2.17E-04 | Unaltered group |
| CLIC3 | -0.86 | 2.41E-04 | Unaltered group |
| CASR | -1.24 | 2.60E-04 | Unaltered group |
| FAR2P1 | 0.98 | 2.61E-04 | Altered group |
| SCEL | -0.82 | 2.74E-04 | Unaltered group |
| CEMIP | -0.86 | 2.86E-04 | Unaltered group |
| SLC18A2 | -0.88 | 2.88E-04 | Unaltered group |
| DHRS9 | -0.94 | 2.94E-04 | Unaltered group |
| HES2 | -0.82 | 3.15E-04 | Unaltered group |
| KCNN4 | -0.84 | 3.29E-04 | Unaltered group |
| CD1B | -0.94 | 4.47E-04 | Unaltered group |
| GRAMD1B | -0.89 | 4.94E-04 | Unaltered group |
| COL2A1 | 1.07 | 5.83E-04 | Altered group |
| MMP1 | -1.15 | 6.15E-04 | Unaltered group |
| VNN1 | -0.87 | 7.51E-04 | Unaltered group |
| MMP28 | -1.01 | 7.51E-04 | Unaltered group |
| HTR2C | 0.88 | 8.36E-04 | Altered group |
| BTNL8 | -0.81 | 8.78E-04 | Unaltered group |
| PADI1 | -1.26 | 9.15E-04 | Unaltered group |
| C2CD6 | 0.86 | 1.03E-03 | Altered group |
| C12ORF56 | 1.09 | 1.13E-03 | Altered group |
| CTSE | -1.2 | 1.18E-03 | Unaltered group |
| COL7A1 | -1.01 | 1.33E-03 | Unaltered group |
| LAMA3 | -0.9 | 1.60E-03 | Unaltered group |
| SDCBP2 | -0.82 | 1.65E-03 | Unaltered group |
| DLX6 | 0.9 | 1.74E-03 | Altered group |
| HOXC13 | 1.28 | 1.77E-03 | Altered group |
| TNC | -0.84 | 1.79E-03 | Unaltered group |
| CRABP1 | 1.05 | 1.87E-03 | Altered group |
| ROS1 | -0.84 | 1.96E-03 | Unaltered group |
| ITPKA | -0.99 | 2.09E-03 | Unaltered group |
| CD1A | -1.05 | 2.27E-03 | Unaltered group |
| LDHC | 0.84 | 2.27E-03 | Altered group |
| HP | -1.27 | 2.34E-03 | Unaltered group |
| PRAME | 1.51 | 2.43E-03 | Altered group |
| FCER2 | -0.81 | 2.47E-03 | Unaltered group |
| MMP10 | -1 | 2.53E-03 | Unaltered group |
| ANXA10 | -1.39 | 2.63E-03 | Unaltered group |
| KRT16 | -1.15 | 2.74E-03 | Unaltered group |
| F5 | -0.88 | 2.84E-03 | Unaltered group |
| SMC1B | 0.86 | 2.86E-03 | Altered group |
| GJB7 | 0.96 | 2.88E-03 | Altered group |
| TMPRSS6 | -1.07 | 2.93E-03 | Unaltered group |
| B3GNT6 | -1.2 | 2.93E-03 | Unaltered group |
| CD1E | -0.82 | 2.95E-03 | Unaltered group |
| MIR31HG | -0.99 | 2.96E-03 | Unaltered group |
| MARCO | -0.83 | 2.97E-03 | Unaltered group |
| DLL3 | 0.99 | 3.56E-03 | Altered group |
| ARL14 | -1.04 | 3.70E-03 | Unaltered group |
| FAT2 | -0.87 | 3.82E-03 | Unaltered group |
| NEFL | -0.82 | 3.96E-03 | Unaltered group |
| SIX2 | 0.96 | 4.01E-03 | Altered group |
| SOHLH2 | 0.89 | 4.15E-03 | Altered group |
| ZG16B | -1.02 | 4.25E-03 | Unaltered group |
| PITX1 | -0.94 | 4.48E-03 | Unaltered group |
| NWD1 | -0.9 | 4.96E-03 | Unaltered group |
| COCH | 0.82 | 5.30E-03 | Altered group |
| MUC5B | -1.43 | 5.35E-03 | Unaltered group |
| TFF2 | -1.3 | 5.40E-03 | Unaltered group |
| CA9 | -1.21 | 5.71E-03 | Unaltered group |
| C6 | -0.88 | 6.13E-03 | Unaltered group |
| ASCL1 | 1.17 | 6.20E-03 | Altered group |
| AKR1B10 | -1.53 | 6.58E-03 | Unaltered group |
| ELF5 | 0.92 | 6.82E-03 | Altered group |
| CHIT1 | -0.84 | 7.18E-03 | Unaltered group |
| DNAH9 | -0.97 | 7.24E-03 | Unaltered group |
| GABRB3 | -0.95 | 7.25E-03 | Unaltered group |
| PCP4 | 1.12 | 7.52E-03 | Altered group |
| VSIG1 | -1.14 | 7.86E-03 | Unaltered group |
| CXCL5 | -0.95 | 8.22E-03 | Unaltered group |
| PAH | 1 | 8.27E-03 | Altered group |
| PTPRT | -0.86 | 8.66E-03 | Unaltered group |
| MUCL3 | -0.98 | 8.68E-03 | Unaltered group |
| PRSS3P2 | -0.98 | 9.28E-03 | Unaltered group |
| AZGP1 | -1.1 | 9.30E-03 | Unaltered group |
| YBX2 | 0.87 | 9.86E-03 | Altered group |
| UCN3 | -0.91 | 0.0101 | Unaltered group |
| GJB3 | -0.89 | 0.0103 | Unaltered group |
| HSD17B2 | -0.98 | 0.0103 | Unaltered group |
| RPS28 | -1.08 | 0.0104 | Unaltered group |
| PRSS1 | -1 | 0.0106 | Unaltered group |
| SYT12 | -0.84 | 0.011 | Unaltered group |
| HBA1 | -0.98 | 0.0119 | Unaltered group |
| CD207 | -0.91 | 0.0127 | Unaltered group |
| LTF | -0.82 | 0.0139 | Unaltered group |
| SAA2 | -0.84 | 0.0167 | Unaltered group |
| TMEM190 | -0.82 | 0.0171 | Unaltered group |
| DRAIC | 0.93 | 0.0178 | Altered group |
| KRT14 | -0.89 | 0.0184 | Unaltered group |
| CYP2C18 | -0.81 | 0.0208 | Unaltered group |
| REG4 | -1.03 | 0.0214 | Unaltered group |
| HOXC11 | 0.93 | 0.0229 | Altered group |
| DKK1 | -0.9 | 0.0238 | Unaltered group |
| SCGB1A1 | -1.19 | 0.0252 | Unaltered group |
| TM4SF4 | -1.06 | 0.0285 | Unaltered group |
| CALCA | 1.14 | 0.0302 | Altered group |
| TFPI2 | -0.89 | 0.0338 | Unaltered group |
| HHIPL2 | -0.9 | 0.0358 | Unaltered group |
| WDR72 | 0.86 | 0.0394 | Altered group |
| KRT6A | -1.02 | 0.0418 | Unaltered group |
| HOXC10 | 0.95 | 0.0418 | Altered group |
| MUC6 | -0.93 | 0.044 | Unaltered group |
| EREG | -0.87 | 0.0466 | Unaltered group |
| CLDN10 | -0.85 | 0.0473 | Unaltered group |
