# Supplementary material for: Comprehensive Analysis of YTH Domain Family in Lung Adenocarcinoma: Expression Profile, Association with Prognostic Value, and Immune Infiltration
Source: Dis Markers. 2021 Aug 26;2021:2789481. doi: 10.1155/2021/2789481 (PMC8420974; doi:10.1155/2021/2789481)
Supplement: Supplementary 4 — Supplementary Table S3: the association between the expression of individual YTH family members and clinical characters in LUAD patients. [file 2789481.f4.docx]

Supplementary Table S3. The association between the expression of individual YTH family members and clinical characters in LUAD patients.

The association between YTHDC1 expression and clinical characters in LUAD patients

| Characteristics | Total(N) | Odds Ratio(OR) | P value |
| --- | --- | --- | --- |
| T stage (T3&T4 vs. T1&T2) | 532 | 0.731 (0.368-1.479) | 0.377 |
| N stage (N1&N2&N3 vs. N0) | 519 | 0.404 (0.237-0.680) | <0.001 |
| M stage (M1 vs. M0) | 386 | 1.582 (0.533-5.024) | 0.424 |
| Pathologic stage (Stage III&Stage IV vs. Stage I&Stage II) | 527 | 0.601 (0.338-1.072) | 0.083 |
| Gender (Male vs. Female) | 535 | 1.208 (0.753-1.948) | 0.434 |
| Age (>65 vs. <=65) | 516 | 0.714 (0.438-1.156) | 0.173 |
| Primary therapy outcome (PR&CR vs. PD&SD) | 446 | 1.244 (0.670-2.302) | 0.487 |
| Smoker (Yes vs. No) | 521 | 1.656 (0.848-3.199) | 0.135 |
| number_pack_years_smoked (>=40 vs. <40) | 369 | 0.700 (0.396-1.224) | 0.213 |

The association between YTHDC2 expression and clinical characters in LUAD patients

| Characteristics | Total(N) | Odds Ratio(OR) | P value |
| --- | --- | --- | --- |
| T stage (T3&T4 vs. T1&T2) | 532 | 0.512 (0.272-0.931) | 0.033 |
| N stage (N1&N2&N3 vs. N0) | 519 | 0.472 (0.298-0.734) | 0.001 |
| M stage (M1 vs. M0) | 386 | 0.614 (0.224-1.546) | 0.324 |
| Pathologic stage (Stage III&Stage IV vs. Stage I&Stage II) | 527 | 0.629 (0.378-1.018) | 0.066 |
| Gender (Male vs. Female) | 535 | 0.816 (0.559-1.184) | 0.287 |
| Age (>65 vs. <=65) | 516 | 0.984 (0.676-1.433) | 0.934 |
| Primary therapy outcome (PR&CR vs. PD&SD) | 446 | 1.217 (0.762-1.985) | 0.420 |
| Smoker (Yes vs. No) | 521 | 0.816 (0.492-1.384) | 0.439 |
| number_pack_years_smoked (>=40 vs. <40) | 369 | 0.701 (0.449-1.078) | 0.110 |

The association between YTHDF1 expression and clinical characters in LUAD patients

| Characteristics | Total(N) | Odds Ratio(OR) | P value |
| --- | --- | --- | --- |
| T stage (T3&T4 vs. T1&T2) | 532 | 0.680 (0.375-1.200) | 0.193 |
| N stage (N1&N2&N3 vs. N0) | 519 | 0.787 (0.521-1.175) | 0.246 |
| M stage (M1 vs. M0) | 386 | 0.999 (0.414-2.230) | 0.998 |
| Pathologic stage (Stage III&Stage IV vs. Stage I&Stage II) | 527 | 0.688 (0.423-1.099) | 0.124 |
| Gender (Male vs. Female) | 535 | 1.113 (0.768-1.616) | 0.572 |
| Age (>65 vs. <=65) | 516 | 0.779 (0.531-1.137) | 0.197 |
| Primary therapy outcome (PR&CR vs. PD&SD) | 446 | 1.176 (0.716-1.963) | 0.528 |
| Smoker (Yes vs. No) | 521 | 1.293 (0.751-2.281) | 0.364 |
| number_pack_years_smoked (>=40 vs. <40) | 369 | 1.385 (0.872-2.216) | 0.170 |

The association between YTHDF2 expression and clinical characters in LUAD patients

| Characteristics | Total(N) | Odds Ratio(OR) | P value |
| --- | --- | --- | --- |
| T stage (T3&T4 vs. T1&T2) | 532 | 0.353 (0.165-0.743) | 0.007 |
| N stage (N1&N2&N3 vs. N0) | 519 | 0.826 (0.485-1.401) | 0.478 |
| M stage (M1 vs. M0) | 386 | 0.752 (0.230-2.465) | 0.637 |
| Pathologic stage (Stage III&Stage IV vs. Stage I&Stage II) | 527 | 0.478 (0.257-0.879) | 0.018 |
| Gender (Male vs. Female) | 535 | 0.662 (0.403-1.080) | 0.100 |
| Age (>65 vs. <=65) | 516 | 0.699 (0.422-1.152) | 0.161 |
| Primary therapy outcome (PR&CR vs. PD&SD) | 446 | 1.192 (0.627-2.279) | 0.594 |
| Smoker (Yes vs. No) | 521 | 0.556 (0.275-1.117) | 0.100 |
| number_pack_years_smoked (>=40 vs. <40) | 369 | 0.648 (0.361-1.152) | 0.142 |

The association between YTHDF3 expression and clinical characters in LUAD patients

| Characteristics | Total(N) | Odds Ratio(OR) | P value |
| --- | --- | --- | --- |
| T stage (T3&T4 vs. T1&T2) | 532 | 1.058 (0.632-1.753) | 0.828 |
| N stage (N1&N2&N3 vs. N0) | 519 | 0.831 (0.569-1.208) | 0.336 |
| M stage (M1 vs. M0) | 386 | 1.041 (0.457-2.267) | 0.922 |
| Pathologic stage (Stage III&Stage IV vs. Stage I&Stage II) | 527 | 1.080 (0.708-1.640) | 0.719 |
| Gender (Male vs. Female) | 535 | 1.387 (0.984-1.969) | 0.064 |
| Age (>65 vs. <=65) | 516 | 0.746 (0.524-1.057) | 0.101 |
| Primary therapy outcome (PR&CR vs. PD&SD) | 446 | 0.947 (0.619-1.459) | 0.804 |
| Smoker (Yes vs. No) | 521 | 1.752 (1.056-2.949) | 0.032 |
| number_pack_years_smoked (>=40 vs. <40) | 369 | 0.688 (0.452-1.036) | 0.076 |
